# Conversion of an inactive xylose isomerase into a functional enzyme by co-expression of GroEL-GroES chaperonins in *Saccharomyces cerevisiae*

**DOI:** 10.1186/s12896-017-0389-7

**Published:** 2017-09-09

**Authors:** Beatriz Temer, Leandro Vieira dos Santos, Victor Augusti Negri, Juliana Pimentel Galhardo, Pedro Henrique Mello Magalhães, Juliana José, Cidnei Marschalk, Thamy Lívia Ribeiro Corrêa, Marcelo Falsarella Carazzolle, Gonçalo Amarante Guimarães Pereira

**Affiliations:** 1 Laboratory of Genomics and Expression, Department of Genetics and Evolution, Institute of Biology, UNICAMP, Campinas, São Paulo, 13083-970 Brazil; 2 CTBE – Brazilian Bioethanol Science and Technology Laboratory, Campinas, SP Brazil

**Keywords:** Xylose isomerase, *Saccharomyces cerevisiae*, Xylose fermentation, GroEL-GroES chaperonins, Ethanol production

## Abstract

**Background:**

Second-generation ethanol production is a clean bioenergy source with potential to mitigate fossil fuel emissions. The engineering of *Saccharomyces cerevisiae* for xylose utilization is an essential step towards the production of this biofuel. Though xylose isomerase (XI) is the key enzyme for xylose conversion, almost half of the XI genes are not functional when expressed in *S. cerevisiae*. To date, protein misfolding is the most plausible hypothesis to explain this phenomenon.

**Results:**

This study demonstrated that XI from the bacterium *Propionibacterium acidipropionici* becomes functional in *S. cerevisiae* when co-expressed with GroEL-GroES chaperonin complex from *Escherichia coli*. The developed strain BTY34, harboring the chaperonin complex, is able to efficiently convert xylose to ethanol with a yield of 0.44 g ethanol/g xylose. Furthermore, the BTY34 strain presents a xylose consumption rate similar to those observed for strains carrying the widely used XI from the fungus *Orpinomyces sp*. In addition, the tetrameric XI structure from *P. acidipropionici* showed an elevated number of hydrophobic amino acid residues on the surface of protein when compared to XI commonly expressed in *S. cerevisiae*.

**Conclusions:**

Based on our results, we elaborate an extensive discussion concerning the uncertainties that surround heterologous expression of xylose isomerases in *S. cerevisiae*. Probably, a correct folding promoted by GroEL-GroES could solve some issues regarding a limited or absent XI activity in *S. cerevisiae*. The strains developed in this work have promising industrial characteristics, and the designed strategy could be an interesting approach to overcome the non-functionality of bacterial protein expression in yeasts.

**Electronic supplementary material:**

The online version of this article (10.1186/s12896-017-0389-7) contains supplementary material, which is available to authorized users.

## Background

Global warming caused by greenhouse gases is becoming a consensus. One of the most efficient ways to avoid further fossil based emissions and capture CO_2_ is through biomass production, with subsequent conversion into biofuels [1]. Recently, the first second-generation (2G) ethanol biorefinery was implemented in Brazil and initiated production [2]. Even though no effort has been spared to optimize the 2G–ethanol production, bottlenecks still need to be overcome, such as the development of a microorganism for efficient pentose (C5-sugar) fermentation, with tolerance to the inhibitors created in the process. The yeast *Saccharomyces cerevisiae* is the microorganism responsible for fermentation in most first-generation (1G) ethanol industries due to its robustness against diverse stresses, high productivity, and elevated ethanol yield. However, this yeast is not capable of naturally consuming C5-sugars [3].

In the course of bioconversion of lignocelluloses, xylose consumption is crucial due to the high percentage of this C5-sugar in its composition [4]. Because *S. cerevisiae* can convert xylulose into ethanol, several works have been and are being developed to obtain a genetically modified strain capable of converting xylose into xylulose. Two xylose conversion pathways are known: the oxidoreductase pathway and the xylose isomerase (XI) pathway. The first appears mainly in fungi and relies on the reduction of xylose to xylitol, followed by the oxidation of xylitol to xylulose through the action of the xylitol reductase (XR) and xylitol dehydrogenase (XDH) enzymes, respectively [3]. The second pathway, although widespread in different organisms, is present mostly in bacteria. It comprises the direct isomerization of xylose to xylulose through the action of the XI enzyme [5]. Both pathways have already been successfully expressed in *S. cerevisiae*. Usually, genes from the XR-XDH pathway can be functionally expressed in *S. cerevisiae*, yet often the genes from XI pathway are not functional when introduced into this yeast.

Pioneering research has tried to express XI from *Escherichia coli*, *Bacillus subtilis*, *Actinoplanes missouriensis,* and *Clostridium thermosulfurogenes* in *S. cerevisiae* without any success [6–8]. The first functional expression registered was achieved with a XI from *Thermus thermophylus*, although with low activity due to optimal temperature for the enzyme action [9]. Years later, the XI from *Piromyces sp*. E2 was also functionally expressed in *S. cerevisiae* generating a yeast with elevated performance [5]. In contrast, several studies tried to develop a pentose consuming *S. cerevisiae* through the expression of bacterial xylose isomerase but were unsuccessful [10–12].

Recently, the *E. coli* chaperonins GroEL-GroES complex where co-expressed in *S. cerevisiae* with xylose isomerase and arabinose isomerase from the same bacteria, which allowed the yeast cells to grow in xylose and arabinose as carbon sources [13]. Expression of the enzymes from *E. coli* without the chaperonins was unable to achieve this effect, which indicates an important role of this complex in enzyme activity.

Several research groups are still seeking new enzymatically functional XI that when expressed in *S. cerevisiae* produce higher ethanol yield with elevated productivity. The ability of *P. acidipropionici* to grow in hydrolyzed materials containing elevated concentrations of xylose has been previously described [14]. The optimum growth of *P. acidipropionici* was registered in anaerobic environment with an optimum temperature and pH around 30 °C and 6.8, respectively, while *S. cerevisiae* has an optimum ethanol production at 30 °C and pH 5.5 [15].

Thus, in this work the xylose consumption pathway of *P. acidipropionici* was analyzed and functionally expressed in an industrial *S. cerevisiae* strain along with the GroEL-GroES complex*.* The influence of the chaperonins in a *S. cerevisiae* strain containing a functional XI from *Orpinomyces sp.* was also studied. Lastly, the uncertainties that surround heterologous expression of xylose isomerases in *S. cerevisiae* are discussed.

## Methods

### Strains and cultivation conditions

Microorganisms and plasmids used in this study are listed in Table 1. *Escherichia coli* strains, used for routine maintenance and preparation of plasmids, were grown in Lysogeny Broth (LB) medium (10 g/L Tryptone, 5 g/L yeast extract, and 10 g/L NaCl, agar 15 g/L when necessary). Antibiotics were added when necessary. *Saccharomyces cerevisiae* strains were grown either in yeast nitrogen base (YNB) medium (6.7 g/L Difco YNB without amino acids) or yeast extract peptone (YP) medium (10 g/L yeast extract, 20 g/L bacto-peptone). *Propionibacterium acidipropionici* was grown in a synthetic medium (PA) as described in Parizzi et al. (2012). Sterile D-glucose or D-xylose was added separately in all media. *E. coli* strains were grown at 37 °C and agitated at 250 rpm when in liquid media. *S. cerevisiae* strains were grown at 30 °C and agitated at 200 rpm for aerobic conditions and at 100 rpm for semi-anaerobic conditions. *P. acidipropionici* was grown under stationary and semi-anaerobic conditions at 30 °C and in batch fermentations under anaerobic conditions at 30 °C, 150 rpm, and pH 6.8. Cell growth was analyzed by OD_600_ determination and samples were taken to determine sugars consumed and products formed.

**Table 1:** Strains and plasmid used in this work

Strain	Genotype (description)	Reference
*E. coli* DH5α	*F– Φ80lacZΔM15 Δ(lacZYA-argF) U169 recA1 endA1* *hsdR17 (rK–, mK+) phoA supE44 λ– thi-1 gyrA96 relA1*	Invitrogen
*P. acidipropionici*	ATCC4875	[45]
JAY270	Industrial *S. cerevisiae* PE-2; *MATa:MATα*	[46]
LVYA1	JAY270; MAT*α*; *ura3Δ*	[40]
BT	LVYA1; pRS426	This work
BTXIPa	LVYA1; pRSXIPa	This work
BTXI2.0	LVYA1; pRSXI2.0	This work
LVY27	LVYA1, *CEN5::pTDH1-xylA-tTDH1*; *gre3Δ*; *CEN2::pADH1-XKS1-tADH1*; *CEN8::pADH1-XKS1-tADH1*; *CEN12::pTDH1-TAL1-tTDH1-pPGK1-RKI1-tPGK1*; *CEN13::pTDH1-TKL1-tTDH1-pPGK1-RPE1-tPGK1*	[40]
LVY65	LVY27, *xylAΔ*, *ura3Δ*	[40]
BTY28	LVY65; pRS426	This work
BTY29	LVY65; pRSXIOrp	This work
BTY30	LVY65; pRSXI2.0	This work
BTY31	LVY65; *CEN5::pPGK-GroEL-tPGK-URA3-pADH1-GroES-tADH1*	This work
BTY32	BTY31; pRS426	This work
BTY33	BTY31; pRSXIOrp	This work
BTY34	BTY31; pRSXI2.0;	This work
Plasmid		
pRS426	ori(f1) - lacZ - T7 promoter - MCS (KpnI-SacI) - T3 promoter - lacI - ori(pMB1) - ampR - ori (2 μm) - *ura3*	[47]
pRSXIPa	pRS426; *pTDH1-XIPa-tTDH*	This work
pRSXI2.0	pRS426; *pTDH1-XI2.0-tTDH*	This work
pRSXIOrp	pRS426; *pTDH1-XIOrp-tTDH*	[40]

### General methods

Genomic DNA from bacteria and yeast strains was extracted with PCI [phenol/chloroform/isoamyl-alcohol (25:24:1)] as previously described [16]. DNA extraction from agarose gels and purification of PCR products were performed using Wizard SV Gel and PCR Clean Up System (Promega). Polymerase chain reaction (PCR) was performed with *Phusion* DNA polymerase (Thermo Fischer Scientific) for construction of the vectors, and with GoTaq polymerase (Promega) for diagnostic purposes. Sanger sequencing was performed in a *3500 Genetic Analyzer* (Applied Biosystems) using “*Big Dye Terminator v3.1 Cycle Sequencing Kit*” (Applied Biosystems) according to the manufacturer’s instructions. DNA was transformed into yeast cells using a standard lithium acetate method [16]. Total protein extraction from yeast strains was performed using Yeast Protein Extraction Reagent (Thermo Fischer Scientific) following the manufacturer’s instructions.

### Cloning of D-xylose isomerase and GroEL-GroES genes for expression in *S. cerevisiae*

The oligonucleotides used in this study are listed in Table 2. The XI gene from *P. acidipropionici* was amplified from genomic DNA using XIO_F and XIO_R. Promoter and terminator regions of constitutive genes (*TDH1*, *ADH1,* and *PGK1*) were amplified from *S. cerevisiae* LVYA1 strain genomic DNA. Genes *xylA*, *groEL,* and *groES* were codon-optimized and synthesized by the company DNA2.0/ATUM. All plasmids used in this work were constructed using *Gibson assembly* [17] and pRS426. GroEL-GroES expression cassette was constructed by Double-Joint PCR and was integrated 516 bp distant from the centromere of chromosome five in *S. cerevisiae* genome through homologous recombination.

**Table 2:** Oligonucleotides used in this work

Oligonucleotide	Sequence
XIO_F	5’ATGGCTGATCTGTGGAACAT3’
XIO_R	5’TCAGGCCTGGGCCAGG3’
XIO_h_pTDH1_F	5’TTCACTAAATTTACACACAAAACAAAATGGCTGATCTGTGGAACAT3’
XIO_h_tTDH1_R	5’TCATTATCCTCATCAAGATTGCTTTATTCAGGCCTGGGCCAG3’
XIOrp_F	5’ATGACTAAAGAATATTTTCCAAC3’
XIOrp_R	5’TTATTGGTACATGGCAACA3’
XIOrp_h_pTDH1_F	5’TCACTAAATTTACACACAAAACAAAATGACTAAAGAATATTTTCCAAC3’
XIOrp_h_tTDH1_R	5’ATTATCCTCATCAAGATTGCTTTATTTATTGGTACATGGCAACA3’
XI2.0_F	5’ATGGCAGATCTCTGGAAT3’
XI2.0_R	5’TTATGCTTGGGCTAAGGC3’
XI2.0_h_pTDH1_F	5’TCACTAAATTTACACACAAAACAAAATGGCAGATCTCTGGAAT3’
XI2.0_h_tTDH1_R	5’ATTATCCTCATCAAGATTGCTTTATTTATGCTTGGGCTAAGGC3’
pTDH1_F	5’TGGTGGATCCATGGCTGATCTGTGGAACAT3’
pTDH1_R	5’TTTGTTTTGTGTGTAAATTTAG3’
pTDH1_h_pRS426_F	5’GATAAGCTTGATATCGAATTCCTGCAGCCCGGGGGATCCAATGTATATGCTCATTTACAC3’
tTDH1_F	5’ATAAAGCAATCTTGATGAGG3’
tTDH1_R	5’CCTGGCCCAGGCCTGAAAGCTTGCGG3’
tTDH1_h_pRS426_R	5’TATTGCTGCCTTTGCAAGGATCCACTAGTTCTAGAGCGGCCGCCACCGCGGTGGAGCTCC3’
GroEL_h_pPGK_F	5’AAGGAAGTAATTATCTACTTTTTACAACAAATATAAAACAATGGCTGCTAAGGACGTTAA3’
GroEL_h_tPGK_R	5’AAAGAAAAAAATTGATCTATCGATTTCAATTCAATTCAATTTACATCATACCACCCATAC3’
GroES_h_pADH1_F	5’TCAAGCTATACCGAGCATACAATCAACTATCTCATATACAATGAACATCAGACCATTGCA3’
GroES_h_tADH1_R	5’CTTATTTAATAATAAAAATCATAAATCATAAGAAATTCGCTTAAGCTTCAACGATAGCCA3’
pPGK_F	5’TACTGTAATTGCTTTTAGTT3’
pPGK_R	5’TGTTTTATATTTGTTGTAAA3’
tPGK_F	5’ATTGAATTGAATTGAAATCG3’
tPGK_h_URA3_R	5’TGGACCATAACTTCGTATAATGTATGCTATACGAAGTTATAAGGCATTAAAAGAGGAGCG3’
pADH1_h_URA3_F	5’ATTTCTATAACTTCGTATAGCATACATTATACGAAGTTATTTCCGGGTGTACAATATGGA3’
pADH1_R	5’TGTATATGAGATAGTTGATTGTATG3’
tADH1_F	5’GCGAATTTCTTATGATTTAT3’
tADH1_R	5’TACAATTGGGTGAAATGGGG3’
URA3loxP_F	5’ATAACTTCGTATAGCATACA3’
URA3loxP_R	5’ATAACTTCGTATAATGTATG3’
URA3_GRE3Δ_F	5’ATATAGAAGCAAATAGTTGTCAGTGCAATCCTTCAAGACGATCACTATAGGGCGAATTGG3’
URA3_GRE3Δ_R	5’GTAAAAATTTATACACATATACAGCATCGGAATGAGGGAAATCTCAAGCTATGCATCCAA3’
Cen2_F	5’TTCAAACTAGGAGTTTGTTGA3’
Cen2_R	5’AAGCTTTCTATTAGTCATTCTTC3’
Check_Cen2_F	5’TGAGACGATTTAGAGTAAGGT3’
Check_Cen2_R	5’GGTGACGACGATATACAG3’
Check_ΔGRE3_F	5’AGCCACATGCGGAAGAAT3’
Check_ΔGRE3_R	5’AAGCGTGGATGACACCAC3’

### Enzyme assays and protein determination

Enzymatic activity of xylose isomerase was determined as described previously [18]. The method was adapted to microplate, and NADH consumption was quantified in spectrophotometer at 340 nm and 30 °C for 15 min. One enzyme unit is defined as the quantity necessary for the conversion of 1 μmol of substrate per minute.

### Bioinformatics tools

The access number of amino acid sequences used in the global alignment and phylogenetic tree construction are listed in Table S1 in Additional file 1. Global alignments among amino acid sequences were carried out using the software MAFFT v.7 [19], with the iterative refinement methods using WSP and consistency scores (G-INS-i), which implements a pipeline combining the WSP and the COFFEE-like score, to evaluate the consistency between a multiple alignment and pairwise alignments. The selection of amino acid substitution models was done using BIC criteria implemented in jModelTest2 [20], and the model that best fit the data was JTT.

The phylogeny was reconstructed using Bayesian analysis implemented on BEAST [21] with two independent rounds of “Metropolis-coupled Markov Chain Monte Carlo” (MCMCMC), in two cold chains and four hot, each analyzed by a million generations and sampled every 100 generations, which resulted in the convergence of the chains.

XI sequences encoded by *P. acidipropionici*, *Orpinomyces sp*., and *Piromyces sp.* were used to find templates for sequence alignment through default BLASTp parameters on the Protein Data bank (PDB). Crystal structures of XI from *Bacillus stearothermophilus* (PDBid:1A0D) were ranked as the best template for molecular modeling procedures based on sequence identity and query coverage quality of the sequences.

For homology modeling of the three-dimensional structures of XI, Modeller 9.16 software [22] was utilized. This software automatically calculates a model containing all non-hydrogen atoms and implements comparative protein structure modeling by satisfaction of spatial restraints. The protein structures where visualized and analyzed by the open-source software of molecular visualization Pymol 1.8 (PyMOL Molecular Graphics System, Version 1.8 Schrödinger, LLC).

### Analytical methods

Cell biomass was calculated by measuring the absorbance at 600 nm in a ULTROSPEC 2000 spectrophotometer UV/visible (Pharmacia Biotech) after appropriate dilution in water. Concentrations of extracellular metabolites were determined by high-pressure liquid chromatography (HPLC) (Waters Alliance Chromatograph), using a refractive index detector (RID) and an ion exclusion column Aminex HPX-87H (Bio-Rad). Samples were filtered through a 0.2 μm filter (Millipore), eluted with 5 mM sulfuric acid at 0.6 ml/min and 35 °C.

## Results

### Why use xylose isomerase from *Propionibacterium acidipropionici*?

**Fig. 1:**
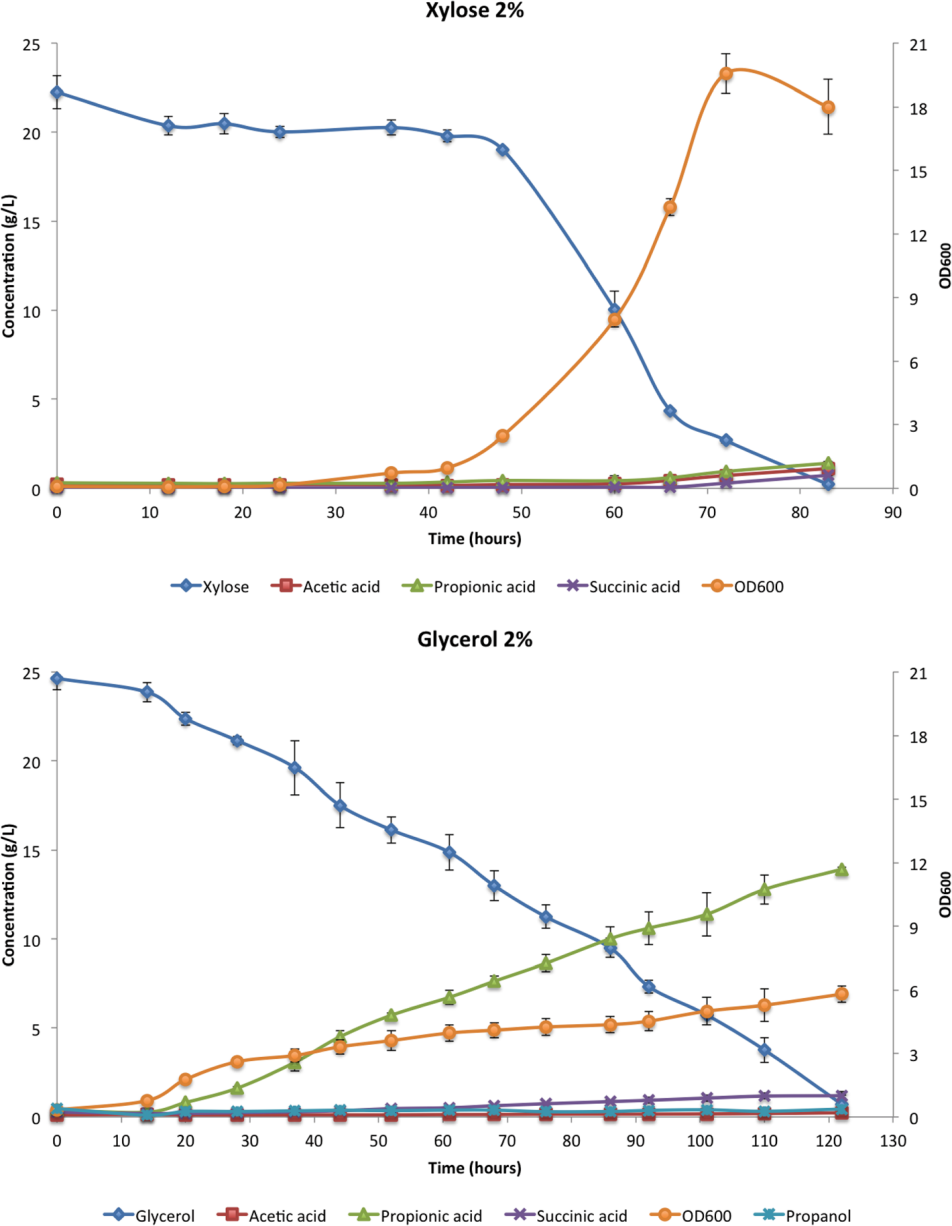
Comparison between *P. acidipropionici* growth, carbon source consumption and product formation in xylose 2% and glycerol 2%. Fermentations were carried out in bioreactors under anaerobic conditions, at 30 °C and pH 6.8. [♦]: carbon source; [■]: acetic acid; [Δ]: propionic acid; [×]: succinic acid; [●]: OD_600_; [✖]: propanol

The bacterium *P. acidipropionici*, as previously mentioned, has physiological characteristics similar to *S. cerevisiae* and is capable of growing in media containing mixtures of xylose and glucose as well as in hydrolyzed biomass [14]. The ability of *P. acidipropionici* to consume xylose was compared in this work to its glycerol consumption. Growth curves, carbon source consumption, and product formation during batch fermentation in 2% xylose and 2% glycerol are represented in Fig. 1. Glycerol is known to be the main carbon source utilized for propionic acid production by *P. acidipropionici* [23]. Compared to fermentations in glycerol, xylose promoted increased biomass production and substrate consumption. Therefore, the obtained results suggest that *P. acidipropionici* presents a xylose conversion pathway with an efficiency comparable to that of its glycerol consumption pathway.

Four putative *xylA* related genes were identified (PACID_03490, PACID_34,060, PACID_34150, PACID_33980). The individual analysis of each gene indicated that only the sequence PACID_03490 had a significant similarity with other prokaryotic XI and, therefore, the study was conducted with this gene. Detailed information about the identified genes can be found in Fig. S1 in Additional file 1.

### Heterologous expression of *P. acidipropionici* D-xylose isomerase in *S. cerevisiae*

*P. acidipropionici* is a bacterium with high GC content in its genome. This characteristic hinders the heterologous expression of proteins from this microorganism in *Saccharomyces cerevisiae* [24]. Therefore, codons were optimized by a third part to an adaptation index more suitable for heterologous expression of the protein in *S. cerevisiae*. Both original and optimized xylose isomerase genes were cloned into the high copy yeast expression vector pRS426 under control of the constitutive promoter *TDH1* and transformed in the *S. cerevisiae* LVYA1 strain, derived from the industrial PE-2, generating the BTXIPa and BTXI2.0 strains. No growth was detected for either strain after cultivation in aerobic conditions (data not shown). Moreover, no XI activity was detected in any of the strains, even though the RNA for the protein was transcribed (Fig. S2 in Additional file 1). In addition, fermentation assays with media supplemented with bivalent cations, such as Mg^2+^, Mn^2+^, and Co^2+^, known as XI cofactors, were performed [25]. Still, no xylose consumption was detected after fermentation under aerobic conditions (data not shown).

### Co-expression of the chaperonins from *E. coli* and *P. acidipropionici* D-xylose isomerase in *S. cerevisiae*

Protein miss-folding is one of the many theories regarding the non-functionality of certain proteins when expressed in *S. cerevisiae*. A work recently developed by Xia et al. (2016) hypothesized that the difference between the chaperonin complexes present in *S. cerevisiae* and *E. coli* was the limiting factor influencing the functional heterologous expression of the *xylA* gene in *E. coli* [13]. In addition, a previous work developed by Guadalupe-Medina et al. (2013) described a *S. cerevisiae* yeast strain containing a bacterial form-II Rubisco that was functional only when co-expressed with the GroEL-GroES chaperonin complex from *E. coli* [26]. These works presented consistent results, showing that the chaperonin complex has a positive influence in heterologous expression of bacterial proteins in yeasts. Therefore, we performed the co-expression of *xylA* from *P. acidipropionici* and GroEL-GroES chaperonin complex in *S. cerevisiae*.

**Fig. 2:**
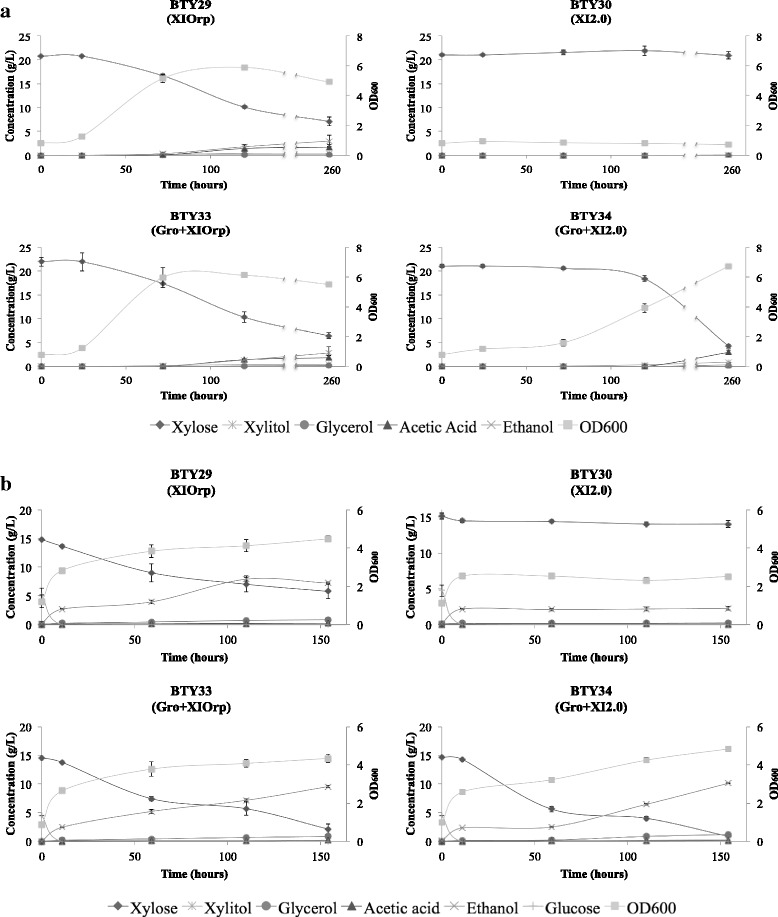
Growth, xylose consumption, and product formation in the developed *S. cerevisiae* strains. [a]: Aerobic growth of strains BTY29, BTY30, BTY33, and BTY34. Fermentations were performed in erlenmeyer flasks at 30 °C and 200 rpm, YNB media without uracil containing xylose 2% as sole carbon source was used. [■]: OD_600_; [♦]: xylose; [×]: ethanol; [*]: xylitol; [●]: glycerol; [Δ]: acetic acid. [b]: Semi-anaerobic growth of BTY29, BTY30, BTY33, and BTY34. Fermentations were performed in SCHOTT flasks at 30 °C and 100 rpm in YNB without uracil media containing a mixture of glucose 0.5% and xylose 2% as carbon source. [■]: OD_600_; [+]: glucose; [♦]: xylose; [×]: ethanol; [*]: xylitol; [•]: glycerol; [**Δ**]: acetic acid

Codon-optimized GroEL-GroES genes from *E. coli* were stably integrated in the *S. cerevisiae* LVY65 strain, generating the strain BTY31 (Table 1). The expression vector with the XI from *P. acidipropionici* (pRSXI2.0) was transformed in the strain LVY65 and BTY31, generating the strains BTY30 and BTY34, respectively (Table 1). For positive and negative controls, the vector containing the XI from *Orpinomyces sp.* (pRSXIOrp) and the empty vector pRS426 were also transformed in both LVY65 and BTY31 strains, generating BTY28 (LVY65, pRS426), BTY29 (LVY65, pRSXIOrp), BTY32 (BTY31, pRS426), and BTY33 (BTY31, pRSXIOrp) (Table 1). Aerobic and semi-anaerobic growth assays were performed. Semi-anaerobic condition was chosen to simulate an industrial environment, where fermentation occurs in large vessels and complete anaerobic conditions are hard to achieve. The obtained results revealed that the BTY34 strain containing *xylA* from *P. acidipropionici* and the chaperonins from *E. coli* was capable of consuming xylose (Fig. 2, a and b, respectively). In fact, BTY34, expressing GroEL-GroES and XI from *P. acidipropionici*, converted xylose into ethanol under semi-anaerobic conditions as efficiently as the BTY29 and BTY33 control strains that contained the *xylA* gene from *Orpinomyces sp*., which is one of the best XI codifying genes described in literature along with the XI from *Piromyces sp.* [27]. The negative controls BTY28 (harboring only the empty vector) and BTY32 (harboring the empty vector and GroEL/GroES) did not display xylose consumption in any of the evaluated conditions (data not shown for BTY28 and BTY32). No growth was detected for the strain carrying only *xylA* from *P. acidipropionici*, BTY30, in all conditions tested. Under aerobic conditions, as expected, low or no ethanol production was detected in the strains capable of consuming xylose: BTY29 (pRSXIOrp), BTY33 (Gro, pRSXIOrp), and BTY34 (Gro, pRSXI2.0).

On the other hand, under semi-anaerobic conditions, BTY34 containing the XI from *P. acidipropionici* not only consumed xylose as well as the positive controls BTY29 (pRSXIOrp) and BTY33 (Gro, pRSXIOrp), but also produced ethanol with comparable efficiency. The ethanol yields observed for BTY34 (Gro, pRSXI2.0) and BTY33 (Gro, pRSXIOrp) where very similar, with values of 0.441 and 0.444 g ethanol/g sugars, respectively (Table 3), which are close to 86% of the theoretical yield (0.51 g ethanol/g sugars). This proves that the enzyme from *P. acidipropionici* in the presence of the chaperonins works as well as the enzyme from *Orpinomyces sp*. When expressed in *S. cerevisiae* and grown under semi-anaerobic conditions. Taking into account that pentose-phosphate pathway genes are overexpressed in the host strain LVY65 along with some other genetic modifications targeting the optimal conversion of C5-sugars into ethanol (Table 1) and the fact that the developed strains did not go through any type of evolution, the ethanol yield obtained in this work becomes more relevant. Evolutionary engineering experiments with BTY34 (Gro, pRSXI2.0), which aim to achieve higher ethanol yield and productivity, are currently underway.

**Table 3:** Product yield during semi-anaerobic fermentation and xylose isomerase activity performed in vitro with crude extract

Strain	Xylitol Yield (g xylitol/g sugars)	Glycerol Yield (g glycerol/g sugars)	Acetic Acid Yield (g acetic acid/g sugars)	Ethanol Yield (g ethanol/g sugars)	Activity (U/mL)
BTY28 (pRS426)	0.000	0.000	0.000	0.161	ND
BTY29 (XIOrp)	0.012	0.043	0.012	0.377	0.073 ± 0.011
BTY30 (XI2.0)	0.005	0.013	0.001	0.198	0.023 ± 0.008
BTY32 (Gro; pRS426)	0.011	0.016	0.000	0.205	ND
BTY33 (Gro;XIOrp)	0.009	0.041	0.010	0.444	0.087 ± 0.006
BTY34 (Gro;XI2.0)	0.014	0.053	0.007	0.441	0.095 ± 0.008

Additionally, enzymatic assays were developed to compare XI activity in the developed strains. Results presented in Table 3 corroborate with the fermentation profile obtained. Strains BTY29 (pRSXIOrp), BTY33 (Gro, pRSXIOrp) and BTY34 (Gro, pRSXI2.0) presented an elevated enzymatic activity in comparison with BTY30 (pRSXI2.0). No activity was detected for BTY28 (pRS426), BTY31 (Gro) or BTY32 (Gro, pRS426).

### Protein modeling of xylose isomerases

**Fig. 3:**
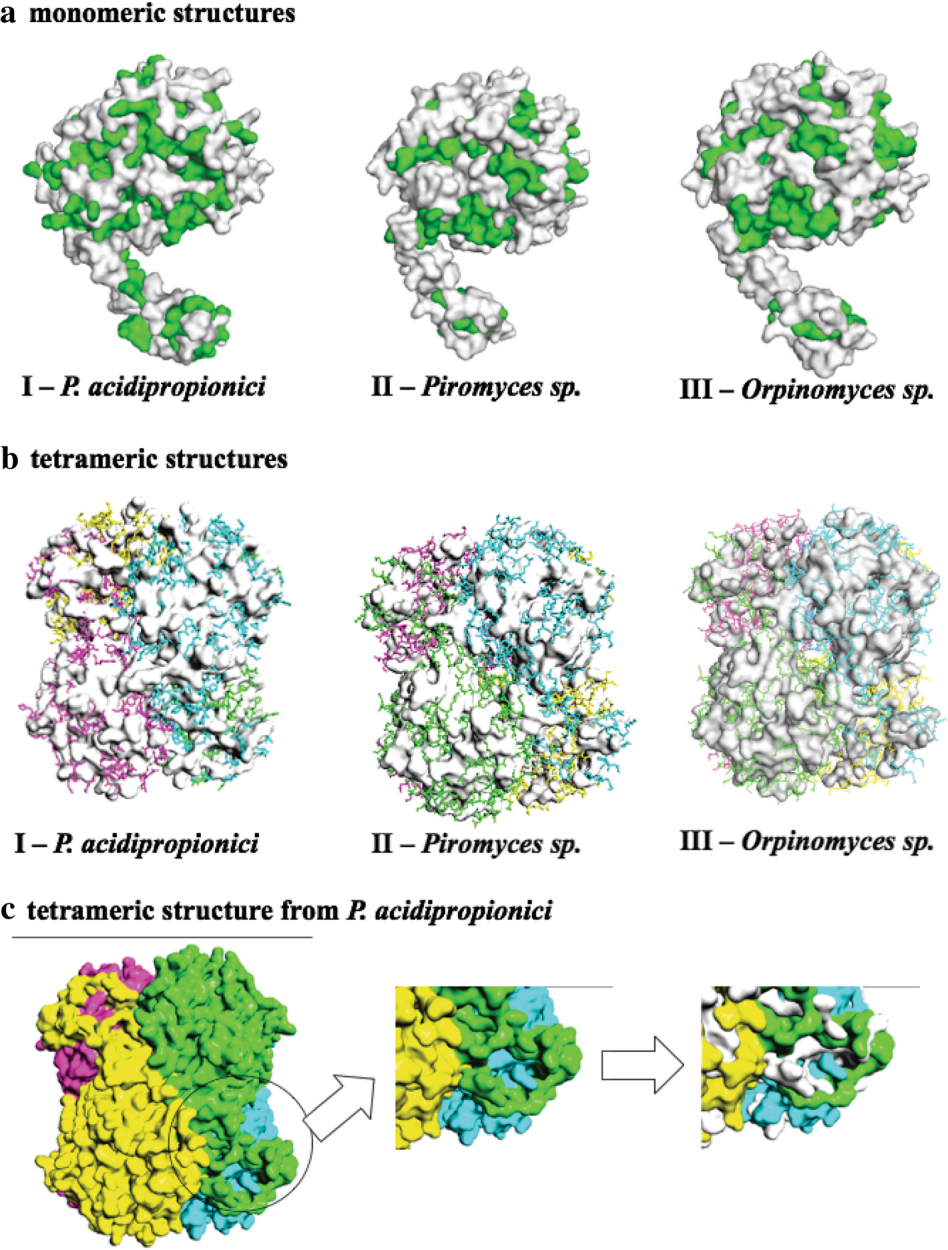
Modeled structures of XIs from *P. acidipropionici* (I), *Piromyces sp*., (II), and *Orpinomyces sp*. (III). [a]: XI monomeric structures, the hydrophobic residues are represented in green and the non-hydrophobic regions in white. [b]: XI tetrameric structures with hydrophobic regions in white surface. [c]: representation of the XI tetrameric structure from *P. acidipropionici* emphasizing the presence of hydrophobic amino acids (white surface in the right box) throughout the region connecting the monomers for the tetrameric structure formation

It is well known that the GroEL-GroES complex interacts with proteins with sizes of 20 to 60 KDa through exposed hydrophobic residues [28]. In that context, the structure of XI proteins from *P. acidipropionici*, *Orpinomyces sp*., and *Piromyces sp.* were modeled in an attempt to elucidate the differences between the hydrophobic amino acids (Fig. 3).

All modeled XI showed a tetrameric quaternary structure with differences in the presence of hydrophobic amino acids on the surface. Interestingly, XI from *P. acidipropionici* showed an elevated number of hydrophobic residues compared to those from *Piromyces sp*. and *Orpinomyces sp*. (Fig. 3b). Likewise, the monomeric structure from the bacterial XI has eleven more hydrophobic residues than the other two analyzed proteins, and the distribution of residues is divergent (Fig. 3a and b). The “tail” area of the protein, where monomers connect for tetramer formation, displays a large number of visually notable differences in the position of hydrophobic residues (Fig. 3c). The observed differences between the hydrophobic residues corroborate the idea that the correct formation of XI from *P. acidipropionici* occurs due to chaperonin interaction.

## Discussion

**Fig. 4:**
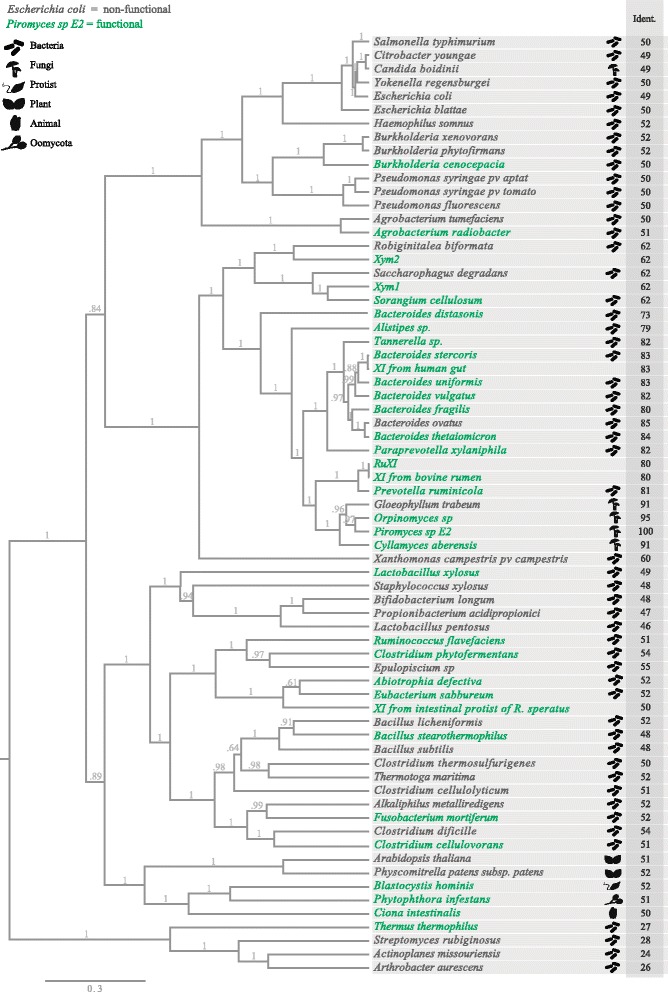
Bayesian phylogenetic tree indicating the evolutionary structure between xylose isomerase proteins expressed in *S. cerevisiae*. Posterior probabilities are indicated in grey above each branch. XI functionally expressed in *S. cerevisiae* are highlighted in green; XI with no function in *S. cerevisiae* are represented in grey; [% Identity]: comprises the percentage of identity of the *xylA* sequences when aligned with *Piromyces sp*. E2; For additional information about the XI sequences used please see Table S1 in Additional file 1

The prospection of new XI proteins for expression in *S. cerevisiae* is usually performed using several criteria, which range from random selection to metagenomics of environments rich in lignocellulose-degrading microorganisms [10, 29, 30]. The innumerous attempts of expressing XI in yeasts are highlighted in Fig. 4.

In some cases, new XIs are chosen considering the similarity with the *Piromyces sp*. protein, as this characteristic may provide higher chances of the prospected XI being functionally expressed in *S. cerevisiae* [31]. As shown in Fig. 4, the similarity between proteins cannot be considered a rule. Not all the XIs described in the literature and successfully expressed in *S. cerevisiae* have high similarity with the protein from *Piromyces sp*. For instance, the XIs from *B. stearothermophylus*, *L. xylosus,* and *T. thermophylus* that have 48%, 49%, and 27% of similarity with *Piromyces sp*., respectively, were functionally expressed.

Phylogenetic proximity of the *xylA* genes being prospected have also been considered for selecting this protein [32]. The phylogenetic tree presented in Fig. 4 compares the phylogenetic distance among most published XI proteins that have been expressed in *S. cerevisiae* to date; notice that the functionality of the protein is not related to the phylogenetic distance between them. The XI proteins from *Piromyces sp*. and *Orpinomyces sp*. are the most studied ones, being known for their high activity when expressed in *S. cerevisiae* [5, 27]. Nevertheless, several distantly related proteins can be functionally expressed in *S. cerevisiae*, e.g. the XI from *Burkholderia cenocepacia*, *Thermus thermophylus*, *Ciona intestinalis*, *Clostridium cellulovorans*, while some closely related proteins have no function at all when introduced in the yeast, such as the XI from the fungus *Gloeophyllum trabeum*, which presents 91% similarity with the XI from *Piromyces sp.*

A total of 69 XI proteins from a range of sources have been expressed in *S. cerevisiae*. These include 55 proteins derived from bacteria, 5 from fungi, 2 from plants, 1 from a protozoa, 1 from a chordate species, 1 from an oomycete, and 4 from metagenomics data. Notably, approximately 49% of these proteins were functionally expressed while the other 51% presented no activity in *S. cerevisiae* with no clear reason (Fig. 5). However, it is possible that a higher number of nonfunctional XIs have already been studied but the negative results were never published.

Even though 51% of *xylA* genes are not functional when introduced in *S. cerevisiae,* not much effort has been put into understanding this issue. Some of the drawn hypotheses include the deficiency of enzymatic cofactors [6], differences in the internal pH of the parental cell and the host cell [6, 9] and the incorrect folding of the protein, which seems to be the most plausible explanation [13, 25]. However, none of these possible causes has been deeply studied.

GroEL-GroES from *E. coli* is a well-known chaperonin complex. They constitute a system that can interact with approximately 250 proteins present in cytosol. The GroEL residue forms a structure similar to a barrel that wraps up proteins. GroES binds to the GroEL-protein complex in one end of the “barrel” forming a structure similar to a “lid”. GroES attachment is ATP dependent and essential for the system to operate [28, 33, 34]. Thus, even though this strategy sounds promising there are some concerns involving the overall performance of the cell because the chaperones will probably bind to several proteins on the cytosol, leading to an unnecessary expenditure of ATP.

In this work, a *S. cerevisiae* strain containing an inactive bacterial xylose isomerase that became functional when co-expressed with GroEL-GroES chaperonin complex from *E. coli* was developed. Results obtained corroborate with a recent study developed by Xia et al. (2016) where the functionality of XI from *E. coli* in *S. cerevisiae* was associated to the chaperonin complex [13]. In addition, the comparison between the modeled XI structures from *Piromyces sp.*, *Orpinomyces sp.,* and *P. acidipropionici* revealed a different pattern in the hydrophobic residues between the bacterial enzyme and the ones from fungi. Considering that the bacterial XI presented an elevated number of exposed hydrophobic residues, located mainly at the tetramer interface, and the fact that interaction between the GroEL-GroES complex and proteins is known to occur through exposed hydrophobic residues [28], it is possible to assume that the GroEL-GroES complex is directly involved in the correct folding of XI from *P. acidipropionici*.

Moreover, the strains developed in this work not only were capable of consuming xylose but also produced ethanol with an elevated yield. The maximum theoretical yield of ethanol in yeasts is considered to be 0.51 g ethanol/g sugar [35],and therefore the yields achieved here are approximately 86% of the theoretical (0.44 g ethanol/g sugar). The best-known described C5 *S. cerevisiae* strains are the ones carrying XI genes from *Piromyces sp*., *Orpinomyces sp*., and *C. phytofermentans* along with several genetic modifications aiming to improve performance of the pentose-phosphate pathway [5, 27, 36].

Previous works have developed several strains containing the mentioned XI, and ethanol yields achieved in the most promising ones were around 84% of the theoretical [37–39]. More recently a C5 strain was developed containing the *Orpinomyces sp*. XI and achieved an ethanol yield of 0.46 g ethanol/g sugar (90% of the theoretical) during anaerobic fermentation [40].

Recently, the importance of eukaryotic chaperonins was discussed in several works. Narayanan et al. (2016) demonstrated that ethanol stress resistance was associated to expression of the eukaryotic protein-folding machine CCT (chaperonin containing t-complex polypeptide) [41]. Also Hou et al., (2016) described a mutation that can cause an up-regulation in chaperone transcriptions in *S. cerevisiae*, leading to enhanced xylose isomerase activity [42]. In addition, a review recently published by Xia et al. (2016) discussed the potential advantages of co-expressing GroEL-GroES complex in yeasts, such as elevated tolerance towards organic inhibitors and temperature changes [43]. Therefore, the fact that the chaperonin complex from *E. coli* is not protein specific can be considered an advantage, especially due to the fact that yeast chaperonins similar to GroEL-GroES are not present in cytosol [44]. Additional research must still be developed to better understand the effect of GroEL-GroES chaperonin complex inside the *S. cerevisiae*.

In summary, this work presented for the first time a *S. cerevisiae* strain co-expressing XI from *P. acidipropionici* and the GroEL-GroES chaperonin complex from *E. coli*. The develop strain BTY34 (Gro, pRSXI2.0) demonstrated an elevated potential for industrial fermentations processes due to its high ethanol yield when compared to a strain containing one of the best XI described in literature.

## Conclusions

Results in this work strongly support the hypothesis that bacterial XI does not always fold correctly inside the yeast. The bacterial *xylA* from *P. acidipropionici*, which was initially not functional in *S. cerevisiae,* when co-expressed with bacterial chaperonins worked with the same efficiency as one of the best XI described in literature. Further studies are required for a complete understanding of the requirements for functional expression of XI in *S. cerevisiae* as well as the dependence of some XIs on chaperonin-assisted-folding.

## Additional file

Additional file 1
**Fig. S1.** Analysis of possible xylose isomerase codifying genes in the genome of *P. acidipropionici*. [a]: possible operon containing the gene PACID_ 03490; [b]: possible operon containing the gene PACID_ 34,060; [c]: possible operon containing the gene PACID_34150 [d]: possible operon containing the gene PACID_33980. The *xylA* candidate genes are represented in green, closely related genes are represented in blue. **Fig. S2.** Agarose gel electrophoresis of *xylA* amplification from cDNA. BT was used as a negative control and RNA translation of *xylA* gene can be observed with amplification of BTXIPa and BTXI2.0 samples. **Table S1.** XI expressed in *Saccharomyces cerevisiae*. (DOC 610 kb)

## Abbreviations

1G: First generation
2G: Second generation
C5-sugar: Pentose sugar
CCT: Chaperonin containing t-complex polypeptide
MFS: Major facilitator superfamily
OD: Optical density
PA: *P. acidipropionici* synthetic media
XDH: Xylitol dehydrogenase
XI: Xylose isomerase
XR: Xylose reductase
YNB: Yeast nitrogen base
