# Supplementary material for: Conversion of an inactive xylose isomerase into a functional enzyme by co-expression of GroEL-GroES chaperonins in Saccharomyces cerevisiae
Source: BMC Biotechnol. 2017 Sep 9;17:71. doi: 10.1186/s12896-017-0389-7 (PMC5591498; doi:10.1186/s12896-017-0389-7)
Supplement: Additional file 1 — Fig. S1. Analysis of possible xylose isomerase codifying genes in the genome of P. acidipropionici. [a]: possible operon containing the gene PACID_ 03490; [b]: possible operon containing the gene PACID_ 34,060; [c]: possible operon containing the gene PACID_34150 [d]: possible operon containing the gene PACID_33980. The xylA candidate genes are represented in green, closely related genes are represented in blue. Fig. S2. Agarose gel electrophoresis of xylA amplification from cDNA. BT was used as a negative control and RNA translation of xylA gene can be observed with amplification of BTXIPa and BTXI2.0 samples. Table S1. XI expressed in Saccharomyces cerevisiae. (DOC 610 kb) [file 12896_2017_389_MOESM1_ESM.doc]

**Additional file 1**


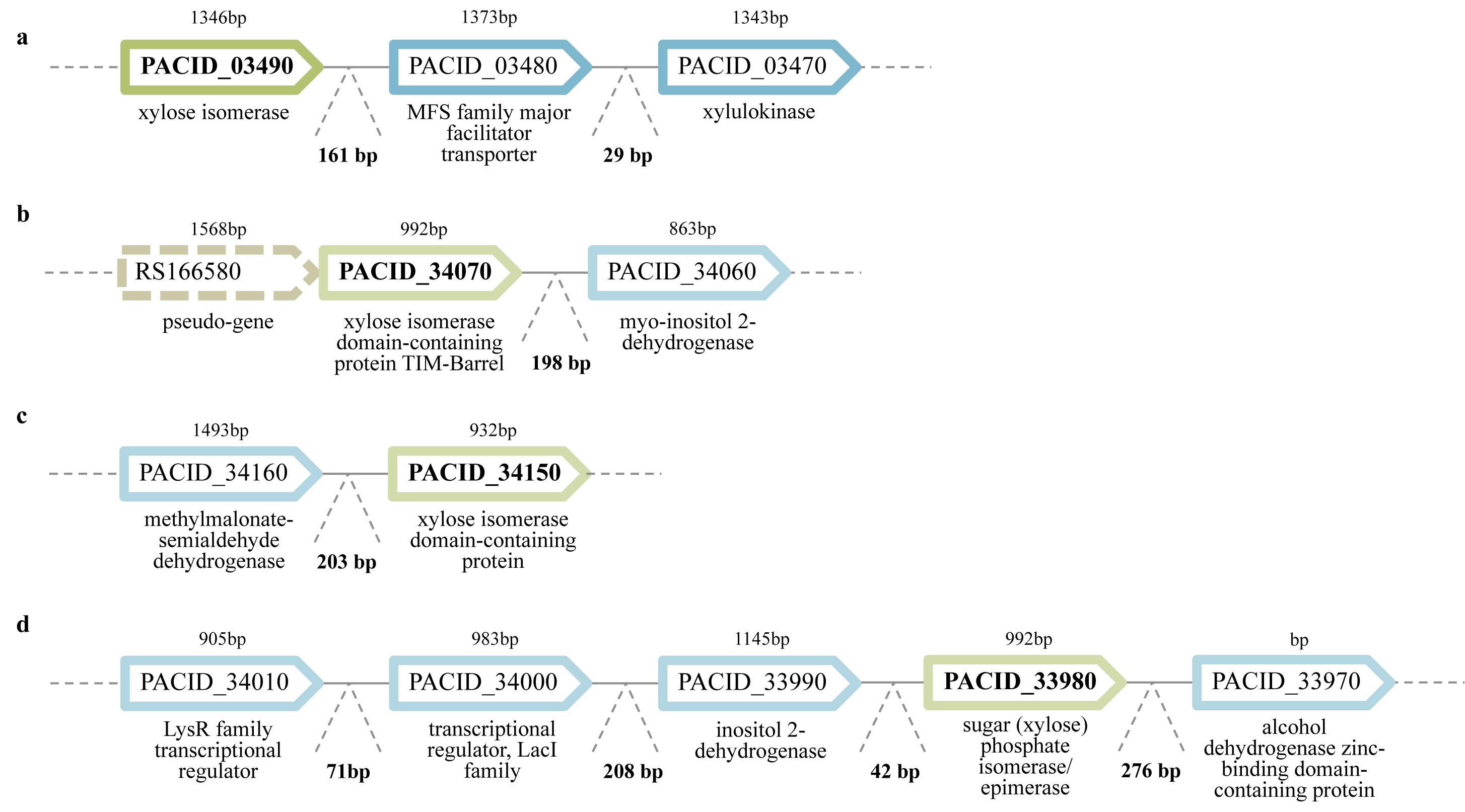


**Figure S1: Analysis of possible xylose isomerase codifying genes in the genome of *P. acidipropionici*. [a]:** possible operon containing the gene PACID_ 03490; **[b]:** possible operon containing the gene PACID_ 34060; **[c]:** possible operon containing the gene PACID_34150 **[d]:** possible operon containing the gene PACID_33980. The *xylA* candidate genes are represented in green, closely related genes are represented in blue. The three genes with low similarity to other XIs (PACID_34060, PACID_34150, and PACID_33980) presented codifying sequences closely positioned, but none had a direct relation to sugar consumption (b, c and d). On the other hand, two other contiguous genes with the same orientation were noted close to *xylA* (PACID_03490). The first one (PACID_03480) codifies a sugar transporter from a major facilitator superfamily (MFS); the second one is the gene that codifies a xylulokinase (PACID_03470) (a). Both genes are directly related to xylose consumption, emphasizing once again that this gene was the one responsible for XI protein formation in *P. acidipropionici*.

**Figure S2: Agarose gel electrophoresis of *xylA* amplification from cDNA.** BT was used as a negative control and RNA translation of *xylA* gene can be observed with amplification of BTXIPa and BTXI2.0 samples.

**TableS1: XI expressed in *Saccharomyces cerevisiae*.** List of XI from innumerous sources that were expressed in the yeast *Saccharomyces cerevisiae*, [+] indicate the enzymes that were functional and [-] indicate the ones that showed no activity in the yeast.

| **XI source / name** | **Functional** | **Optimized** | **Identity with *Piromyces sp*.** | **Group** | **Sequence**  **access number** | **Reference** |
| --- | --- | --- | --- | --- | --- | --- |
| *Abiotrophia defectiva* | + | Yes | 52% | Bacteria | - | [1] |
| *Actinoplanes missouriensis* str. DSM43046 | - | No | 24% | Bacteria | P12851 | [2] |
| *Agrobacterium radiobacter* K84 (A. tumeaciens) | + | No | 51% | Bacteria | B9JAF5 | [3] |
| *Agrobacterium tumefaciens* C58 | - | No | 50% | Bacteria | Q8U7G6 | [4] |
| *Alistipes sp.* HGB5 | + | Yes | 79% | Bacteria | E4MCK9 | [5] |
| *Alkaliphilus metalliredigens* QYMF | - | Yes | 52% | Bacteria | A6TUP7 | [6] |
| *Arabdopsis thaliana* | - | No | 51% | Plant | Q9FKK7 | [4] |
| *Arthrobacter aurescens* TC1 | - | Yes | 26% | Bacteria | A1RAY0 | [7] |
| *Bacillus licheniformis str.* ATCC14580 | - | No | 52% | Bacteria | P77832 | [4] |
| *Bacillus stearothermophylus* (*Geobacillus stearothermophylus strain* T-6) | + | Yes | 48% | Bacteria | Q09LW9 | [8] |
| *Bacillus subtilis str.* 168 | - | No | 48% | Bacteria | P0CI80 | [2] |
| *Bacteroides distasonis* str. ATCC8503 (*Parabacteroides distasonis*) | + | Yes | 73% | Bacteria | A6LA39 | [9] |
| *Bacteroides fragilis* str. DSM2151 | + | Yes | 80% | Bacteria | Q5LCV9 | [7] |
| *Bacteroides ovatus* ATCC8483 | - | Yes | 85% | Bacteria | A7LXH2 | [9] |
| *Bacteroides stercoris* HJ-15 | + | No | 83% | Bacteria | G1AUD0 | [10] |
| *Bacteroides thetaiotaomicron* ATCC 29148 | + | Yes | 84% | Bacteria | Q8A9M2 | [11] |
| *Bacteroides uniformis* ATCC8492 | + | Yes | 83% | Bacteria | A7UZG5 | [12] |
| *Bacteroides vulgatus* ATCC8482 | + | Yes | 82% | Bacteria | A6L792 | [5] |
| *Bifidobacterium longum* MG1 | - | No | 48% | Bacteria | G1AUD1 | [10] |
| *Blastocystis hominis* | + | Yes | 52% | Protist | D8MBL6 | [13] |
| *Burkholderia cenocepacia* J2315 | + | No | 50% | Bacteria | B4ENA5 | [14] |
| *Burkholderia phytofirmans* PsJN | - | Yes | 52% | Bacteria | B2T929 | [7] |
| *Burkholderia xenovorans* LB400 | - | No | 52% | Bacteria | Q13RB8 | [4] |
| *Candida boidinii* | - | Yes | 49% | Fungi | - | [13] |
| *Ciona intestinalis* | + | Yes | 50% | Animal | F6WBF5 | [7] |
| *Citrobacter youngae* ATCC29220 | - | - | 49% | Bacteria | D4BII5 | [15] |
| *Clostridium cellulolyticum* H10 | - | Yes | 51% | Bacteria | B8I1T2 | [6] |
| *Clostridium cellulovonans* 743B | + | No | 51% | Bacteria | D9SR73 | [16] |
| *Clostridium difficile* M120 | - | Yes | 54% | Bacteria | FN665653.1 | [7] |
| *Clostridium phytofermentans* ISDg | + | Yes | 54% | Bacteria | A9KN98 | [4] |
| *Clostridium thermosulfurogenes* (*Thermoanaerobacterium thermosulfurigenes*) | - | No | 50% | Bacteria | P19148 | [17] |
| *Cyllamyces aberensis* | + | Yes | 91% | Fungi | HV778520.1 | [7] |
| *Epulopiscium sp. N.t. morphotype B* | - | Yes | 55% | Bacteria | - | [6] |
| *Escherichia blattae* str. DSM4481 (*Shimwellia blattae*) | - | - | 50% | Bacteria | I2B3Z9 | [15] |
| *Escherichia coli* K12 | - | No | 49% | Bacteria | P00944 | [18] |
| *Eubacterium sabbureum* str. DSM3986 (*Lachnoanaerobaculum saburreum*) | + | No | 52% | Bacteria | E6LP05 | [19] |
| *Fusobacterium mortiferum* ATCC9817 | + | Yes | 52% | Bacteria | C3W9E8 | [7] |
| *Gloeophyllum trabeum* | - | Yes | 91% | Fungi | - | [13] |
| *Haemophilus somnus* 129pt | - | Yes | 52% | Bacteria | Q0I346 | [7] |
| *Lactobacillus pentosus* str. DSM 20314 | - | No | 46% | Bacteria | A0A0R1FPH9 | [4] |
| *Lactobacillus xylosus* (*Lactococcus lactis* DSM20175) | + | No | 49% | Bacteria | Q9CFG7 | [20] |
| *Orpinomyces sp.* ukk1 | + | No | 95% | Fungi | B7SLY1 | [21] |
| *Paraprevotella xylaniphila* YIT11841 | + | Yes | 82% | Bacteria | F3QTK4 | [5] |
| *Physcomitrella patens subsp. patens* | - | Yes | 52% | Plant | A9T7Y3 | [7] |
| *Phytophthora infestans* T30-4 | + | Yes | 51% | Oomycota | D0NA42 | [6] |
| *Piromyces sp.* E2 | + | No | 100% | Fungi | Q9P8C9 | [22] |
| *Prevotella ruminicola* TC2-24 (*Bacteroides ruminicola*) | + | Yes | 81% | Bacteria | R9R7L8 | [9] |
| *Propionibacterium acidipropionici* | - | Yes | 47% | Bacteria |  | This work |
| *Pseudomonas fluorescens* (99% identity) | - | No | 50% | Bacteria | Q3KDW0 | [23] |
| *Pseudomonas syringae pv. aptata* str. DSM 50252 | - | No | 50% | Bacteria | F3J2B1 | [4] |
| *Pseudomonas syringae pv. tomato* 323 | - | - | 50% | Bacteria | A0A099SLK6 | [15] |
| *Robiginitalea biformata* str. DSM 15991 | - | No | 62% | Bacteria | A4CGS8 | [4] |
| *Ruminococcus flavefaciens* 007c | + | Yes | 51% | Bacteria | W7UZA6 | [6] |
| RuXI | + | No | 80% | - | - | [24] |
| *Saccharophagus degradans* str. DSM 17024 | - | No | 62% | Bacteria | Q21HR5 | [4] |
| *Salmonella typhimurium* LT2 | - | No | 50% | Bacteria | Q8ZL90 | [4] |
| *Sorangium cellulosum* str. So ce56 | + | No | 62% | Bacteria | A9FY79 | [23] |
| *Staphylococcus xylosus* str. DSM 20266 | - | No | 48% | Bacteria | P27157 | [4] |
| *Streptomyces rubiginosus* str. ATCC1927 | - | No | 28% | Bacteria | P24300 | [25] |
| *Tannerella sp.* 6_1_58FAACT1 | + | Yes | 82% | Bacteria | G9S7T9 | [5] |
| *Thermotoga maritima* MSB8 | - | Yes | 52% | Bacteria | Q9X1Z5 | [7] |
| *Thermus thermophilus* HB8 | + | No | 27% | Bacteria | P26997 | [26] |
| *Xanthomonas campestris pv. campestris* str. DSM 3586 | - | No | 60% | Bacteria | Q8P9T9 | [4] |
| XI from bovine rumen | + | Yes | 80% | - | - | [13] |
| XI from human gut | + | Yes | 83% | - | - | [13] |
| XI from intestinal protozoa of *Reticulitermes speratus* | + | No | 50% | - | - | [27] |
| Xym1 - XI gene from uncultured bacterium | + | No | 62% | Bacteria | AEG75765.1 | [28] |
| Xym2 - XI gene from uncultured bacterium | + | No | 62% | Bacteria | AEG75766.1 | [28] |
| *Yokenella regensbungei* ATCC43003 | - | - | 50% | Bacteria | G9Z1E8 | [15] |

**References**

1. Subbian E, Zhang X. Pentose fermentation by a recombinant microorganism. 2010. p. 62. Available from: https://www.google.com/patents/WO2012009272A2?cl=de

2. Amore R, Wilhelm M, Hollenberg CP. The fermentation of xylose - an analysis of the expression of *Bacillus* and *Actinoplanes* xylose isomerase genes in yeast. Appl. Microbiol. Biotechnol. 1989;30:351–7. Available from: http://link.springer.com/10.1007/BF00296623

3. Kim DM, Choi S-H, Ko BS, Jeong G-Y, Jang H-B, Han J-G, et al. Reduction of PDC1 expression in *S. cerevisiae* with xylose isomerase on xylose medium. Bioprocess Biosyst. Eng. 2012;35:183–9. Available from: http://link.springer.com/10.1007/s00449-011-0638-4

4. Brat D, Boles E, Keller M, Wiedemann B. Prokaryotic xylose isomerase for the construction of xylose fermenting yeasts. 2009. p. 21. Available from: https://www.google.ch/patents/US20110269180

5. Peng B, Huang S, Liu T, Geng A. Bacterial xylose isomerases from the mammal gut *Bacteroidetes* cluster function in *Saccharomyces cerevisiae* for effective xylose fermentation. Microb. Cell Fact. 2015;14:70. Available from: http://www.microbialcellfactories.com/content/14/1/70

6. Subbian E, Zhang X. Pentose fermentation by a recombinat microorganism. 2010. p. 63. Available from: https://www.google.com/patents/WO2012024046A2?cl=en11

7. Teunissen AWRH, De Bont JAM. Xylose isomerase genes and their use in fermentation of pentose sugars. 2009. p. 16. Available from: https://www.google.com/patents/US20110318790

8. Klassen P, Laan JM Van Der, Gielesen BEM, Suylekom GP. A pentose sugar fermenting cell. 2009. p. 57. Available from: https://www.google.com/patents/WO2009109634A1?cl=deFl

9. Hector RE, Dien BS, Cotta M a, Mertens J a. Growth and fermentation of D-xylose by *Saccharomyces cerevisiae* expressing a novel D-xylose isomerase originating from the bacterium *Prevotella ruminicola* TC2-24. Biotechnol. Biofuels. 2013;6:84. Available from: http://www.ncbi.nlm.nih.gov/pubmed/23721368

10. Ha S-J, Kim SR, Choi J-H, Park MS, Jin Y-S. Xylitol does not inhibit xylose fermentation by engineered *Saccharomyces cerevisiae* expressing xylA as severely as it inhibits xylose isomerase reaction in vitro. Appl. Microbiol. Biotechnol. 2011;92:77–84. Available from: http://link.springer.com/10.1007/s00253-011-3345-9

11. Mert MJ, la Grange DC, Rose SH, van Zyl WH. Engineering of *Saccharomyces cerevisiae* to utilize xylan as a sole carbohydrate source by co-expression of an endoxylanase, xylosidase and a bacterial xylose isomerase. J. Ind. Microbiol. Biotechnol. 2016; Available from: http://link.springer.com/10.1007/s10295-015-1727-1

12. Klassen P, Laan JM Van Der, Gielesen BEM, Suylekom GP. A pentose sugar fermenting cell. 2009. p. 75. Available from: http://www.google.co.in/patents/WO2009109633A1?cl=und

13. Alvizo O, Miller MG, Mijts B, Meshulam-Simon G, McDaniel R. Pentose fermentation by a recombinant microorganism. 2013. p. 84. Available from: https://www.google.ch/patents/US20150337340

14. de Figueiredo Vilela L, de Mello VM, Reis VCB, Bon EP da S, Gonçalves Torres FA, Neves BC, et al. Functional expression of *Burkholderia cenocepacia* xylose isomerase in yeast increases ethanol production from a glucose–xylose blend. Bioresour. Technol. 2013;128:792–6. Available from: http://www.sciencedirect.com/science/article/pii/S0960852412015167

15. Hitz WD, Qi M, Rush SE, Tao L, Viitanem V, Yang J, et al. Expression of xylose isomerase activity in yeast. 2013. p. 131. Available from: https://www.google.com/patents/US20140178954?hl=pt-PT

16. Ota M, Sakuragi H, Morisaka H, Kuroda K, Miyake H, Tamaru Y, et al. Display of *Clostridium cellulovorans* xylose isomerase on the cell surface of *Saccharomyces cerevisiae* and its direct application to xylose fermentation. Biotechnol. Prog. 2013;29:346–51. Available from: http://doi.wiley.com/10.1002/btpr.1700

17. Moes CJ, Pretorius IS, van Zyl WH. Cloning and expression of the *Clostridium thermosulfurogenes* D-xylose isomerase gene (*xyLA*) in *Saccharomyces cerevisiae*. Biotechnol. Lett. 1996;18:269–74. Available from: http://link.springer.com/10.1007/BF00142943

18. Sarthy A V, McConaughy BL, Lobo Z, Sundstrom J a, Furlong CE, Hall BD, et al. Expression of the *Escherichia coli* xylose isomerase gene in *Saccharomyces cerevisiae*. Appl. Environ. Microbiol. 1987;53:1996–2000. Available from: http://www.pubmedcentral.nih.gov/articlerender.fcgi?artid=204047&tool=pmcentrez&rendertype=abstract

19. Dragovic Z, Gamauf C, Reisinger, Kettling U. Pentose fermenting microorganisms. 2014. Available from: https://www.google.ch/patents/US20140377813?hl=de&cl=en

20. Rönnow B, Andersen TH, Sibbesen O. Microorganism Expressing Xylose Isomerase. 2009. p. 54. Available from: https://www.google.ch/patents/US20110244525

21. Madhavan A, Tamalampudi S, Ushida K, Kanai D, Katahira S, Srivastava A, et al. Xylose isomerase from polycentric fungus *Orpinomyces*: Gene sequencing, cloning, and expression in *Saccharomyces cerevisiae* for bioconversion of xylose to ethanol. Appl. Microbiol. Biotechnol. 2009;82:1067–78.

22. Kuyper M, Harhangi HR, Stave AK, Winkler A a., Jetten MSM, De Laat WT a M, et al. High-level functional expression of a fungal xylose isomerase: The key to efficient ethanolic fermentation of xylose by *Saccharomyces cerevisiae*? FEMS Yeast Res. 2003;4:69–78.

23. Hou J, Shen Y, Jiao C, Ge R, Zhang X, Bao X. Characterization and evolution of xylose isomerase screened from the bovine rumen metagenome in *Saccharomyces cerevisiae*. J. Biosci. Bioeng. 2016;121:160–5. Available from: http://linkinghub.elsevier.com/retrieve/pii/S1389172315002078

24. Bao X, Shen Y, GE R. Nucleic acid molecule for encoding xylose isomerase and xylose isomerase encoded by the nucleic acid molecule. 2012. Available from: https://www.google.com/patents/US20120225452

25. Gárdonyi M, Hahn-Hägerdal B. The *Streptomyces rubiginosus* xylose isomerase is misfolded when expressed in *Saccharomyces cerevisiae*. Enzyme Microb. Technol. 2003;32:252–9. Available from: http://linkinghub.elsevier.com/retrieve/pii/S0141022902002855

26. Walfridsson M, Bao X, Anderlund M, Lilius G, Bülow L, Hahn-Hägerdal B. Ethanolic fermentation of xylose with *Saccharomyces cerevisiae* harboring the *Thermus thermophilus xylA* gene, which expresses an active xylose (glucose) isomerase. Appl. Environ. Microbiol. 1996;62:4648–51. Available from: http://www.ncbi.nlm.nih.gov/pubmed/8953736

27. Onishi T, Tada N, Yasutani N, Katahira S, Ishida N, Nagura R. Method for producing ethanol using recombinat yeast. 2014. p. 80. Available from: https://encrypted.google.com/patents/US20160002674?cl=ar

28. Parachin N, Gorwa-Grauslund MF. Isolation of xylose isomerases by sequence- and function-based screening from a soil metagenomic library. Biotechnol. Biofuels. BioMed Central Ltd; 2011;4:9. Available from: http://www.biotechnologyforbiofuels.com/content/4/1/9
